# Perioperative Left Ventricular Assist Device Use in Patients With Reduced Ejection Fraction Reduces Cardiac Surgery-Associated Acute Kidney Injury

**DOI:** 10.7759/cureus.57248

**Published:** 2024-03-30

**Authors:** Ujjawal Kumar, Zain Khalpey

**Affiliations:** 1 School of Clinical Medicine, University of Cambridge, Cambridge, GBR; 2 Department of Cardiothoracic Surgery, HonorHealth, Scottsdale, USA

**Keywords:** left ventricular assist device, impella, adult cardiac surgery, cardiorenal protection, acute kidney injury

## Abstract

Background

Cardiac surgery may precipitate acute kidney injury (AKI), particularly in patients with poor baseline cardiac function. This is thought to be due to intraoperative renal hypoperfusion, which results in increased morbidity and mortality. This study evaluated the perioperative use of the Impella LD (Abiomed, Danvers, MA) left ventricular assist device (LVAD) in the prevention of postoperative AKI in patients with reduced left ventricular ejection fraction (LVEF) undergoing cardiac surgery.

Methods

A retrospective analysis was performed at Northwest Medical Center, Tucson, AZ, USA, on patients undergoing valve surgery, coronary artery bypass grafting (CABG), or both by a single surgeon. Those with preoperative LVEF ≤35% and preoperative serum creatinine ≥1 mg/dL were included and segregated based on intraoperative LVAD implantation. Postoperative renal function was assessed using serum creatinine levels and KDIGO (Kidney Disease Improving Global Outcomes) criteria to define AKI.

Results

Twenty-three patients were enrolled. There were no significant differences in age, demographics, baseline characteristics, or comorbidities between the treatment (n = 12) and the control group (n = 11). In the treatment group, 8% developed AKI by POD#7, while 64% of controls did. The treatment group had a significantly lower mean creatinine change from POD#0-7 (0.07 vs. 0.59, p = 0.02). However, there was no significant difference between groups in the mean creatinine change from baseline to discharge (0.46 vs. 0.42, p = 0.47).

Conclusions

Our study suggests that intraoperative Impella implantation may reduce the incidence of early postoperative AKI. LVAD implantation is an approach to increase and ensure adequate end-organ (renal) perfusion and can improve postoperative recovery without dialysis requirements. Additional studies are required to understand its protective effects during the perioperative period fully.

## Introduction

Open cardiac surgery carries inherent risks of various postoperative complications, with acute kidney injury (AKI) being a particularly concerning complication that impacts prognostic outcomes. Patients with reduced baseline cardiac function who undergo open cardiac surgery are at a higher risk of inadequate organ perfusion and subsequent AKI. Despite advancements in AKI management, it remains a significant contributor to poor short- and long-term prognosis following cardiac surgery [1]. Identifying high-risk individuals and implementing nephroprotective strategies may help reduce the occurrence of this serious complication and improve surgical outcomes. Perioperative use of left ventricular assist devices (LVADs), such as the Impella LD (Abiomed, Danvers, MA), could potentially mitigate AKI and prevent the need for continuous renal replacement therapy (CRRT) in this high-risk patient population.

The incidence of cardiac surgery-associated AKI (CSA-AKI) ranges between 22% and 36% [2-4], with severe cases requiring renal replacement therapy in up to 4% of patients [5]. ​​ Postoperative AKI is associated with increased morbidity and mortality, both in the short term and up to 10 years postoperatively [6]. Severe CSA-AKI requiring CRRT confers mortality rates as high as 70% (odds ratio (OR) = 4.2) [7,8]. CSA-AKI is independently associated with increased rates of myocardial infarction and bleeding [9]. Recent works, such as the Door-To-Unload in STEMI Pilot Trial [10], have demonstrated that partial hemodynamic support with percutaneous LVADs, such as the Impella CP, can ensure adequate organ perfusion during high-risk cardiac procedures, leading to reduced reperfusion injury and smaller infarct sizes. Infarct size is known to be a prognostic indicator of future heart failure and mortality, and therefore Impella CP use had a role in improving patient outcomes.

We suggest that LVADs could play a role in mechanically unloading the left ventricle during weaning from cardiopulmonary bypass (CPB), thereby improving organ perfusion. This is particularly relevant in patients with poor baseline cardiac function, as intraoperative transient hypoperfusion may precipitate irreversible kidney damage. LVADs may help mitigate AKI by improving kidney function, as indicated by smaller decreases in serum creatinine (SCr), associated with lower mortality [11]. The LVAD of interest in our study was the Impella LD. The Impella LD is a specific type of LVAD that incorporates a small rotary pump to facilitate blood flow. It is implanted below the aortic valve in the left ventricular outflow tract (LVOT), with a cannula passing through the aortic valve and an output port in the ascending aorta. The Impella LD is increasingly utilized to treat cardiogenic shock and improve organ perfusion in patients with poor left ventricular ejection fraction (LVEF) postoperatively to prevent ischemic tissue damage, the setting in which it was evaluated in our study.

In this study, we evaluated the utility of perioperative Impella LD implantation to improve renal perfusion and reduce the incidence of AKI following open cardiac surgery, particularly in patients with poor baseline cardiac function.

This study was previously presented at the Society of Thoracic Surgeons Coronary Conference in Miami, on the 3rd and 4th of June 2023.

## Materials and methods

A single-surgeon retrospective analysis was conducted at Northwest Medical Center, Tucson, AZ, USA, on 23 patients undergoing cardiac surgery from 2019-2021 (isolated coronary artery bypass grafting, isolated valve surgery, or both).

Patient inclusion

Patients in this study were selected based on their preoperative characteristics. As a part of the preoperative assessment for cardiac surgery, various imaging studies, including transthoracic echocardiography (TTE), were conducted to measure the LVEF. In addition, blood samples were collected before surgery; SCr levels served as an indicator of the preoperative renal function. Patients with a preoperative LVEF ≤35%, indicating a high risk of developing CSA-AKI, and SCr ≥ 1 mg/dL (no baseline renal dysfunction) were included. Patients were stratified into two groups: the control group (patients who underwent cardiac surgery without Impella LD placement, n = 11) and the treatment group (patients with an Impella LD device placed intra-operatively during cardiac surgery, n = 12).

Operative procedure

**Figure 1:**
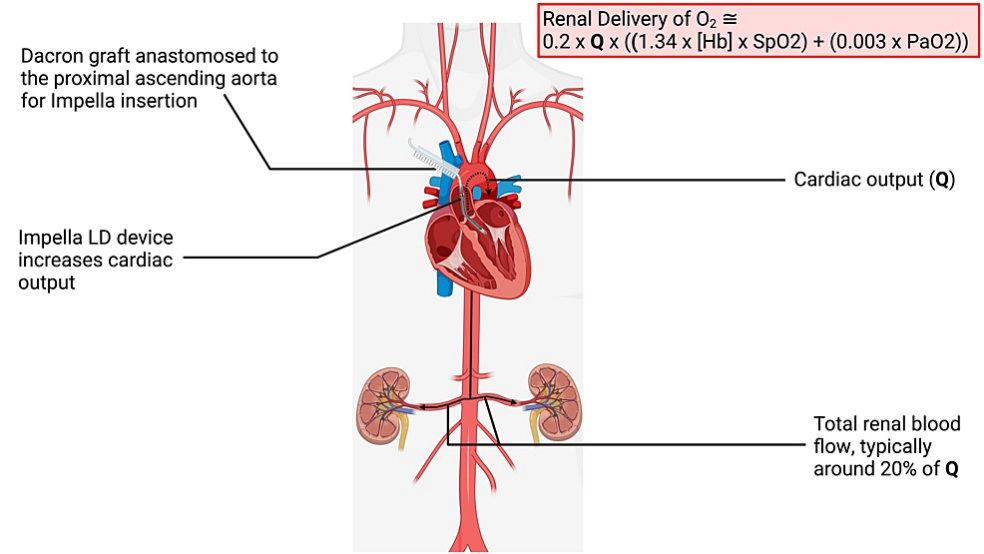
The implanted Impella LD device across the aortic valve, with delivery graft. Impella LD device implanted across the aortic valve, as described, using the Dacron graft. By unloading the heart and increasing the cardiac output, Q, the device increases total renal blood flow. It therefore increases renal oxygen delivery, known to be directly proportional to renal blood flow. Figure created with BioRender.com. Image Credits: BioRender original icons library.

Impella LD implantation differs from percutaneous Impella devices (Impella 2.5, CP, 5.0, 5.5, RP). A surgical cut-down approach with supraclavicular tunneling [12] was used (Figure 1). An aortotomy was made proximal to the cross-clamp, ≥7 cm distal to the aortic valve plane, and a vascular graft (10 mm x 15 cm; Hemashield; Getinge Maquet, Wayne, NJ) was anastomosed to the ascending aorta. An incision was made superolateral to the sternal notch to access the supraclavicular fossa. The graft was tunneled behind the manubrium and sternoclavicular joint, exiting the skin between the sternocleidomastoid muscle heads.

**Figure 2:**
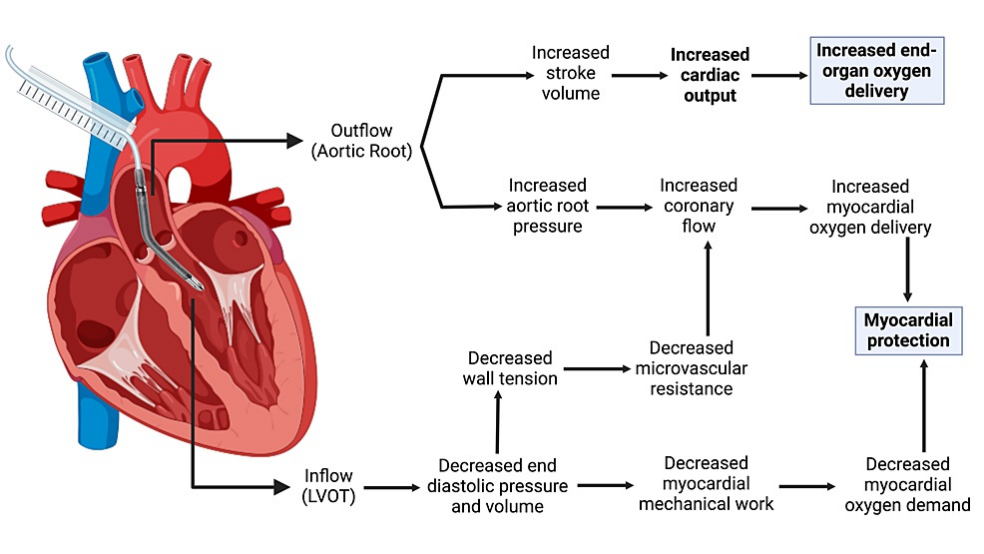
Effects of Impella LD use on cardiac physiology. The Impella LD device has a series of effects on cardiac physiology, with two aims: improvement of end-organ perfusion and myocardial protection. Figure created with BioRender.com. Image Credits: BioRender original icons library.

The primed Impella LD device was advanced through the graft and across the aortic valve while the patient remained on CPB. Under TEE guidance, the device was advanced to position the intake port approximately 3.5cm into the LVOT, and the graft was secured in the supraclavicular fossa. During CPB weaning, the pump was de-aired and gradually increased to full flow (4.4L/min, 30,000rpm, P8). The physiological effects of the Impella LD system while in use are shown in Figure 2. The Impella device was later explanted under local anesthesia.

Data collection and analysis

Serial SCr levels were analyzed daily from the day of cardiac surgery (POD#0) until discharge. AKI was defined using SCr at POD#7 using the KDIGO (Kidney Disease Improving Global Outcomes) criteria, as shown in Table 1 [13]. Patient demographics, length of admission, and other parameters were recorded. The results were stored in a secure document. No patient identifiers were used. Statistical analyses (Chi-squared, T-tests) were conducted to compare groups. IRB approval (WIRB Study#1275376; IRB#20200195; Feb 5, 2020) and patient consent were obtained for relevant treatments and retrospective data interpretation. A p-value of 0.05 was considered the significance threshold, as is conventional.

**Table 1:** KDIGO criteria for staging and classification of AKI. Our study used the KDIGO criteria [13] to identify patients who developed acute kidney function (AKI) by POD#7. KDIGO (Kidney Disease: Improving Global Outcomes) is an international organization that develops widely used clinical practice guidelines.

Stage	Renal function	Urine output
1	1.5–1.9 x baseline; ≥0.3 mg/dL increase	<0.5mL/kg/h for 6-12 h
2	2.0–2.9 x baseline	<0.5mL/kg/h for ≥12 h
3	3.0 x baseline; ≥4.0 mg/dL increase; initiation of RRT; decrease in eGFR to ≤ 35 mL/min/1.73m^2^	<0.3mL/kg/h for ≥24 h

## Results

Twenty-three patients were enrolled in this study and segregated into two experimental groups: the treatment group (n = 12, mean age 71 ± 7.1 years, male:female ratio 10:2), and the control group (n = 11, mean age 71 ± 8.3 years, male:female ratio 7:4). The two groups did not differ significantly in their mean age, other demographics, baseline characteristics, or comorbidities (Table 2). Notably, the preoperative serum creatinine was measured in both groups, with no significant difference between the treatment group and the control group (Table 2), indicating a similar baseline renal function in patients of both study groups. This validated our conclusions from any subsequent differences in serum creatinine and renal function, as these could confidently be attributed to the postoperative course and whether Impella support was provided.

**Table 2:** Demographics and baseline characteristics for patients included in this study. No significant differences between the treatment and control groups in any demographic or clinical characteristics. Categorical variables (sex: male, preoperative use of IABP, previous cardiac surgery, COPD requiring medical therapy, diabetes mellitus requiring insulin, hypertension, emergent surgery) are expressed in this table as N (%). Continuous variables (age, STS risk: Renal Failure Score, Thakar Risk Score, preoperative LVEF, preoperative serum creatinine) are expressed in this table as mean ± SD. A result was considered significant if p ≤ 0.05. P-values were calculated for each variable by comparing the treatment group to the control group. STS: Society of Thoracic Surgeons, LVEF: left ventricular ejection fraction, IABP: intraaortic balloon pump, COPD: chronic obstructive pulmonary disease

Variable (mean ± SD, or n (%))	Total	Treatment group (perioperative Impella LD)	Control group (no intervention)	p-value
Number	23 (100%)	12 (100%)	11 (100%)	
Age, years	71.0 ± 7.5	71.0 ± 7.1	71.0 ± 8.3	0.970
Sex, male	17 (74%)	10 (83%)	7 (64%)	0.292
STS risk: Renal Failure Score	2.516 ± 2.20	1.877 ± 0.86	3.283 ± 3.03	0.139
Thakar Risk Score	4.26 ± 4.94	4.35 ± 6.09	4.16 ± 3.58	0.929
Preoperative LVEF (%)	30.22 ± 9.6	31.25 ± 12.5	29.09 ± 5.4	0.592
Preoperative serum creatinine, mg/dL	1.20 ± 0.33	1.25 ± 0.36	1.15 ± 0.30	0.246
Preoperative use of IABP	2 (8.7%)	0 (0.0%)	2 (18.2%)	0.134
Previous cardiac surgery	4 (17.4%)	3 (25.0%)	1 (9.1%)	0.337
COPD requiring medical therapy	4 (17.4%)	2 (16.7%)	2 (18.2%)	0.928
Diabetes mellitus requiring insulin	1 (4.3%)	1 (8.3%)	0 (0.0%)	0.350
Hypertension	15 (65.2%)	7 (58.3%)	8 (72.7%)	0.491
Emergent surgery	6 (26.1%)	2 (16.7%)	4 (36.4%)	0.304

Following surgery, Impella support was weaned according to the local hospital protocol with improving cardiac function until postoperative day 7 (POD#7) when the Impella device was explanted. Across these first seven postoperative days, serum creatinine levels were measured daily (mean values shown in Table 3), allowing for assessment of kidney function and diagnosis of AKI at postoperative day 7. The peak serum creatinine concentration reached, as well as the serum creatinine concentration at discharge, are also shown in Table 3.

**Table 3:** Comparison of daily serum creatinine concentration to the serum creatinine concentration on the day of surgery (postoperative day 0). No significant change in serum creatinine from postoperative day 0 to any of the first seven days following surgery, peak value, or to discharge in the treatment group. Significant increases from postoperative day 0 to days 6 (p = 0.039) and 7 (p = 0.032) in the control group. A significant increase from postoperative day 0 to the peak value reached throughout hospitalization (p = 0.047). No significant changes from the day of surgery to any other day in the control group. No significant differences in serum creatinine concentration between the treatment and control groups on any of the days. A result was considered significant if p ≤ 0.05. P-values were calculated for each day’s serum creatinine concentration by conducting a t-test between the serum creatinine concentrations on that particular day and comparing them to the serum creatinine concentrations on the day of surgery.

Postoperative day	Treatment group: serum creatinine (mg/dL)	p-value: ∆SCr from POD#0	Control group: serum creatinine (mg/dL)	p-value: ∆SCr from POD#0	p-value: group comparison
0	1.28	N/A	1.16	N/A	0.350
1	1.33	0.786	1.27	0.489	0.775
2	1.44	0.399	1.45	0.113	0.986
3	1.40	0.518	1.50	0.105	0.672
4	1.40	0.498	1.52	0.108	0.622
5	1.33	0.803	1.65	0.061	0.231
6	1.36	0.634	1.77	0.039	0.159
7	1.35	0.675	1.84	0.032	0.118
Peak	2.00	0.204	1.97	0.047	0.968
Discharge	1.71	0.284	1.57	0.321	0.807

**Figure 3:**
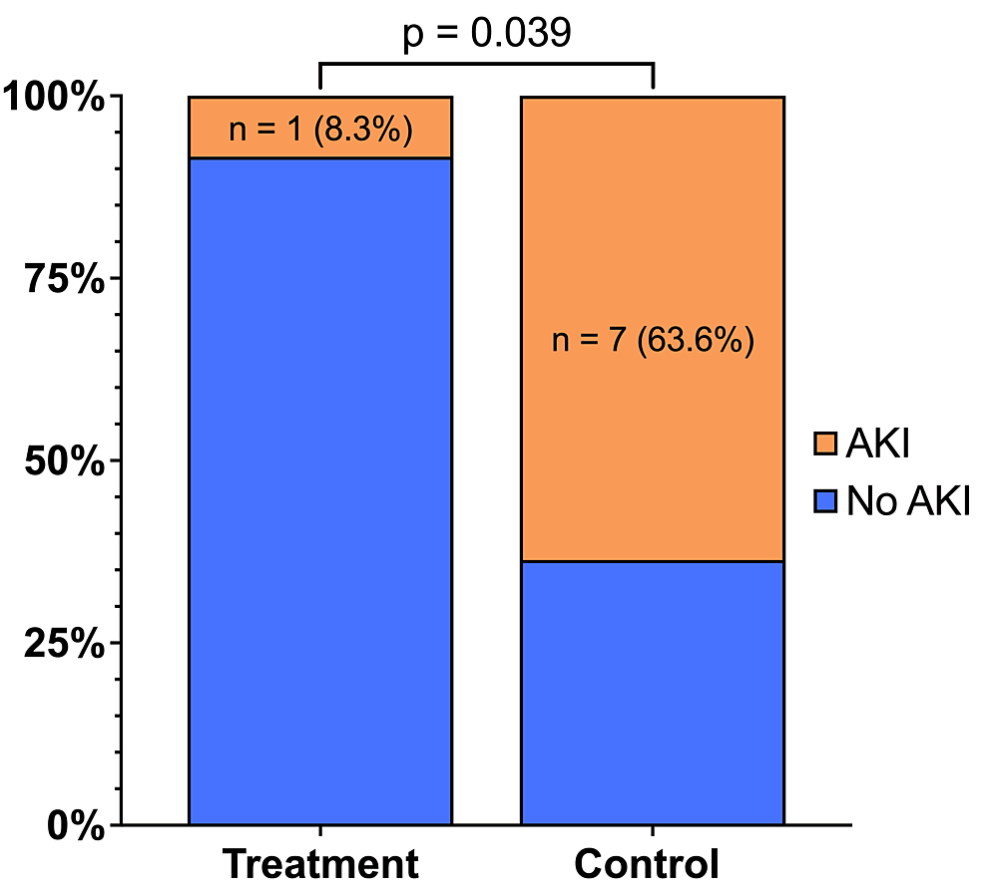
Comparison of AKI Incidence between groups. The incidence of an AKI at postoperative day 7 (POD#7) was compared between the treatment and control groups. A significantly lower (p = 0.039) proportion of patients in the treatment group (n = 1, 8.3%) developed AKI compared to the control group (n = 7, 63.6%). A result was considered significant if p ≤ 0.05. P-values were calculated for each variable by comparing the treatment group to the control group. AKI: acute kidney injury, POD: postoperative day

Using the serum creatinine concentration measured on the seventh postoperative day, we found that a significantly lower proportion of patients in the treatment group developed an AKI compared to the control group (Figure 3). The relative risk of AKI in the treatment group compared to the control group was therefore 0.131.

**Figure 4:**
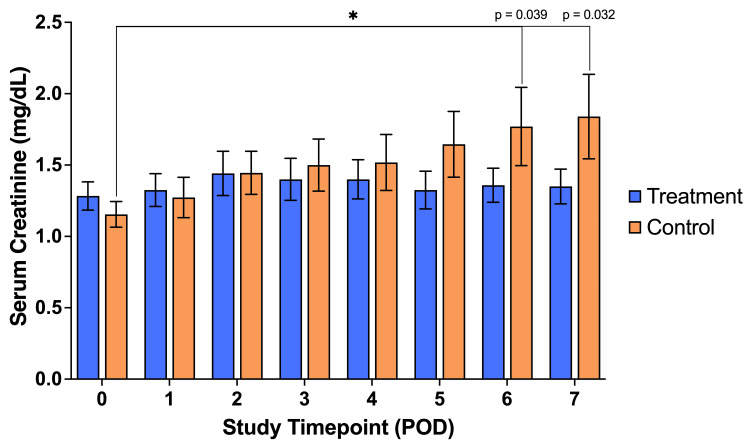
Serial mean serum creatinine concentration (SCr) values. Serial mean serum creatinine concentration (SCr) values across our patient population; significant increases in the control group from baseline on postoperative days (PODs) #6 and 7. No other significant differences were observed. A result was considered significant if p ≤ 0.05. P-values were calculated for each variable by comparing the treatment group to the control group.

Throughout the seven days, the serum creatinine concentration was not significantly different between the treatment group and the control group. In the treatment group, there were no significant changes in serum creatinine concentration within the first seven postoperative days when comparing each consecutive day to the day of surgery (POD#0). In the control group, there were no significant changes in the first five days postoperatively. However, on the sixth and seventh postoperative days, the increase in serum creatinine concentrations reached statistical significance (Figure 4).

There was no significant difference between the peak creatinine levels reached throughout their admission by patients in both groups. However, in the control group, the increase in serum creatinine concentration from the day of surgery to the peak value amounted to a significantly greater increase in the control group (Table 3). There were no significant changes from the day of surgery to discharge in the serum creatinine concentration in either group. In addition, when the serum creatinine concentration at discharge was compared between study groups, there was no significant difference between the treatment and control groups, implying no significant difference in renal function at the point of hospital discharge.

Next, to assess renal protection resulting from LVAD support, any postoperative changes in kidney function were compared between groups. This was done by comparing the change in serum creatinine concentration (∆SCr) from the day of surgery (POD#0) to each successive day up to the seventh postoperative day, as well as to the peak SCr reached throughout hospitalization and at discharge (Table 4).

**Table 4:** Group comparison of changes in serum creatinine from the day of surgery (POD#0) to successive time points. Significantly greater increase in serum creatinine from the day of surgery to the fifth (p = 0.021), sixth (p = 0.018), and seventh (p = 0.012) postoperative days. The change from the day of surgery was not significantly different between groups at any other time point within the first seven postoperative days or indeed at discharge. POD: postoperative day A result was considered significant if p ≤ 0.05. P-values were calculated for each variable by comparing the treatment group to the control group.

Outcome	Treatment group (mg/dL)	Control group (mg/dL)	p-value
∆SCr POD#0 - 1	0.04	0.12	0.308
∆SCr POD#0 - 2	0.16	0.29	0.387
∆SCr POD#0 - 3	0.12	0.35	0.173
∆SCr POD#0 - 4	0.12	0.36	0.155
∆SCr POD#0 - 5	0.04	0.49	0.021
∆SCr POD#0 - 6	0.08	0.60	0.018
∆SCr POD#0 - 7	0.07	0.67	0.012
∆SCr POD#0 - Peak	0.75	0.82	0.916
∆SCr POD#0 - Discharge	0.48	0.42	0.911

**Figure 5:**
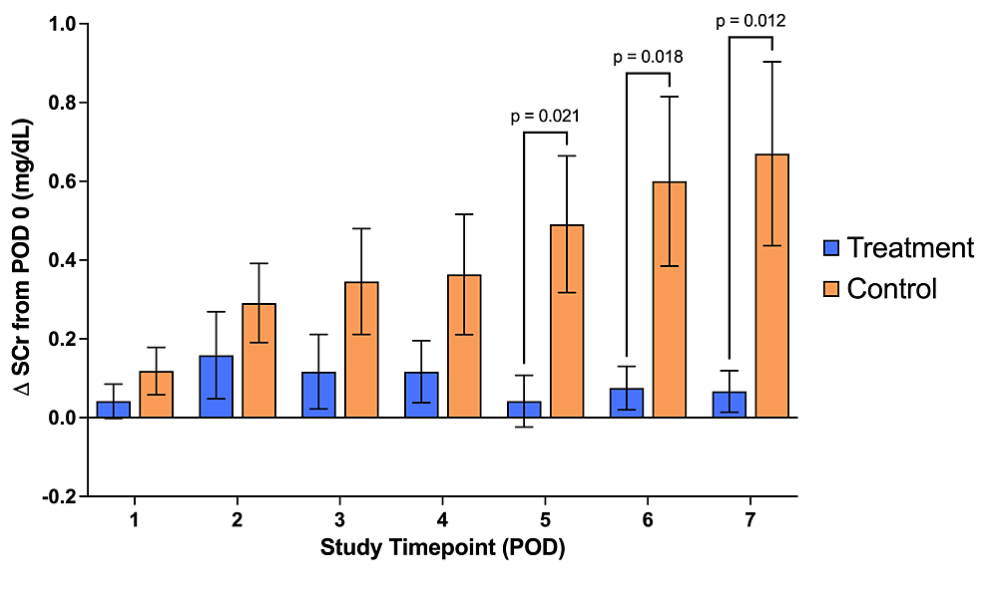
Consecutive change in serum creatinine from baseline. Consecutive change in serum creatinine from baseline (∆SCr); significant differences between groups on the fifth, sixth, and seventh postoperative days (PODs). No significant difference between groups on POD#1, 2, 3, or 4. A result was considered significant if p ≤ 0.05. P-values were calculated for each variable by comparing the treatment group to the control group.

By comparing the serum creatinine changes from the day of surgery to any of the first four postoperative days, there was no significant difference between the treatment and control groups. However, there was a significantly lower ∆SCr in the treatment group than in the control group in the fifth, sixth, and seventh postoperative days (Figure 5). Comparing the change from the preoperative SCr to the peak SCr reached during hospitalization, this was lower in the treatment group than the control group, although not significantly. Likewise, there was no significant difference between the two groups when the mean creatinine change was compared for the duration of hospitalization, i.e., comparing creatinine change from POD#0 to the day of discharge.

**Figure 6:**
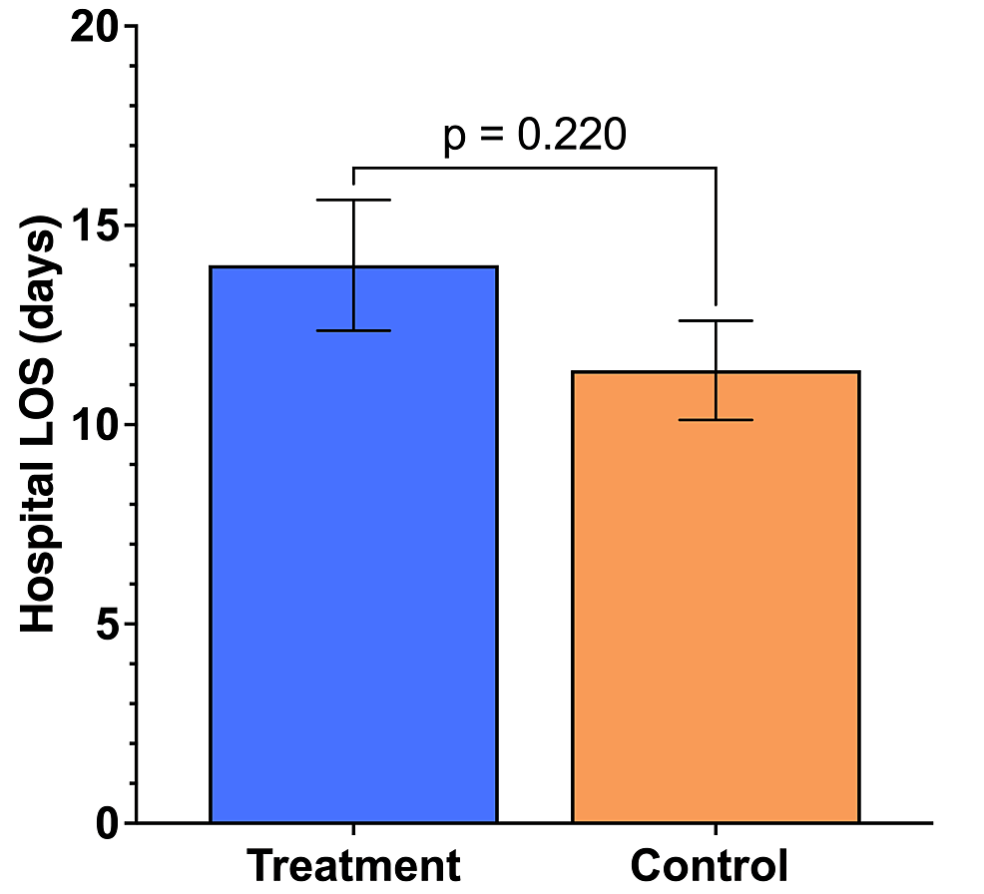
Comparison of length of hospital admission between the groups. No significant difference in the length of hospital admission in the treatment and control groups. A result was considered significant if p ≤ 0.05. P-values were calculated for each variable by comparing the treatment group to the control group.

Lastly, the length of hospital admission was not significantly different between the treatment group and the control group (Figure 6), despite the additional care requirements in patients receiving Impella LD treatment. These additional requirements include care of the tunneling site and/or anticoagulation management for mechanical circulatory support.

## Discussion

AKI is a common complication of cardiac surgery, impacting patient outcomes [6]. Aside from impacting patient outcomes and prognosis, the financial impact of AKI following cardiac surgery is significant. It is estimated that the mean total index hospitalization cost is $77,178 for patients with AKI following cardiac surgery, compared to $38,820 for those without. At the US national level, this correlates with an annual increase in index hospitalization costs associated with CSA-AKI of around $1 billion [14]. Therefore, developing strategies to minimize renal damage, decrease AKI occurrence, and potentially eliminate the need for renal replacement therapy can significantly enhance patient care after cardiac surgery and health system performance.

Existing nephroprotective strategies include promoting recovery after a previous episode, employing minimally invasive surgical techniques, reducing CPB use and nephrotoxic substances (e.g., radiocontrast dyes), and optimizing cardiovascular function through the administration of inotropic support, fluids, and pressors [15]. However, as these approaches may not be universally applicable, we aimed to evaluate the effectiveness of perioperative LVAD support as an additional tool to expand treatment options and improve patient outcomes.

In our study, we found the intraoperative implantation of the Impella LD to significantly reduce AKI incidence, giving us confidence in its merit as a strategy to reduce CSA-AKI in patients with reduced baseline LVEF. To gain further confidence in our conclusions, further studies are currently underway by our group to elucidate the role of LVADs in mitigating CSA-AKI. These include a much larger sample size of patients, undergoing a range of cardiac surgical procedures, although all with preoperative left ventricular dysfunction, defined as LVEF ≤35%.

We assessed the impact of Impella usage on kidney function by analyzing serial serum creatinine levels. Our data revealed a significant increase in SCr levels from baseline to the fifth, sixth, and seventh postoperative days in the control group, compared to the treatment group. This suggests that the Impella LD device effectively protected kidney function between days five and seven following cardiac surgery, providing therapeutic benefits. Interestingly, there was no significant difference between the groups in the first four postoperative days, implying that this early period following surgery may not heavily contribute to AKI development by the seventh postoperative day. It was encouraging that in the treatment group, the change from baseline to peak serum creatinine was not a significant increase. This shows that the increased renal perfusion due to LVAD use meant that kidney function was protected throughout admission across the study group.

While the mean discharge SCr did not differ significantly between groups, this does not provide a comprehensive view. It must be noted that patients who developed AKI received renal replacement therapy, resulting in normalized serum creatinine levels before discharge. However, in long-term follow-up data, we anticipate a notable difference in morbidity and mortality attributable to RRT administration. This distinction is not apparent by examining serum creatinine levels at discharge alone.

While the length of hospital stay (LOS) was not significantly different (slightly longer mean LOS in the treatment group), this minor distinction is attributable to additional care involved in managing LVAD patients, such as anticoagulation management and care of the tunneling site, which are unrelated to renal function or cardiac recovery but necessary due to the intervention [16]. Importantly, these factors are now known to affect patient outcomes or overall prognosis.

Further investigation is warranted to assess the long-term effects of perioperative treatments on renal function. As a part of routine follow-up after cardiac surgery, patients were seen in the clinic two weeks post-discharge or earlier if their condition worsened. It is essential to evaluate the future renal function of patients in both the treatment and control groups to examine any sustained impacts and identify potential long-term differences. In addition, expanding the patient cohort and including individuals with varying comorbidities would enhance the reliability of our findings and conclusions. For instance, instead of exclusively including patients with LVEF ≤35%, we could incorporate individuals with different levels of LV dysfunction, utilizing widely accepted criteria such as mild LV dysfunction (41% to 50%), moderate LV dysfunction (31% to 40%), or severe LV dysfunction (≤30%) [17].

Another avenue for further investigation is the impact of perioperative LVAD use in patients with known diabetes mellitus. Patients with diabetes controlled with oral hypoglycemic agents (DM-oral) and those with insulin-controlled diabetes (DM-insulin) are both at a higher risk of AKI following cardiac surgery than the non-diabetic population (adjusted odds ratios: DM-oral = 1.26, DM-insulin = 3.92, [18]). As a high-risk patient population for CSA-AKI, these individuals would particularly benefit from additional interventions to mitigate the risk of AKI.

In our study, we identified patients with preoperative LVEF ≤35% as high risk of developing AKI, due to the risk of renal hypoperfusion. We found that established systems, such as the STS risk score for operative risk, as well as scoring systems designed specifically to predict CSA-AKI risk using preoperative characteristics [19-21], did not successfully predict future AKI in the respective patients. These models have well-recognized limitations, such as the homogeneity of patients included in their development, the exclusion of high-risk cardiac surgical procedures, and those patients with preoperative renal dysfunction, and the fact that data often only includes a single center. Within our research group, we are currently developing an artificial intelligence-based risk stratification system that will be trained using a multi-center, diverse large dataset, and therefore we suggest that this system will have much greater predictive utility than currently available systems.

Our vision for the future involves delivering personalized treatment and perioperative protocols to patients, aiming to decrease morbidity and mortality while enhancing overall outcomes. In summary, the findings of our study suggest that LVADs may warrant consideration for routine use in high-risk patients undergoing cardiac surgery to protect against CSA-AKI. This could ultimately improve patient outcomes by reducing AKI rates, which will have the benefit of reducing the number of patients requiring CRRT, and its associated risks and costs. Routine elective use of Impella LD in high-risk cardiac surgical patients may also mitigate secondary risks of AKI, such as bleeding and future myocardial infarctions, among others. We foresee this will initially be an institutional practice, and with more data and corroboration with other centers, a potentially more widely accepted practice.

## Conclusions

Patients with poor baseline cardiac function, defined as a preoperative LVEF≤ 35%, are at high risk of CSA-AKI, due to intraoperative renal hypoperfusion. LVADs, such as the Impella LD, improve cardiac output and renal oxygen delivery and, as this study showed, can significantly reduce AKI rates in this high-risk patient population. We therefore advocate for the routine use of temporary LVADs in the perioperative management of such high-risk cardiac surgery patients as a part of a pragmatic approach to protect patients against AKI. This study contributes to the ongoing dialogue on the utility of LVADs in cardiac surgery, proposing a paradigm shift toward more personalized, mechanistically informed interventions to prevent CSA-AKI. This study has shown the therapeutic potential of LVADs in reducing the burden of AKI, underscoring the importance of integrating advanced technological solutions into cardiac care, paving the way for improved patient outcomes and reduced healthcare costs.
